# Genipin-Crosslinked Chitosan Gels and Scaffolds for Tissue Engineering and Regeneration of Cartilage and Bone

**DOI:** 10.3390/md13127068

**Published:** 2015-12-11

**Authors:** Riccardo A. A. Muzzarelli, Mohamad El Mehtedi, Carlo Bottegoni, Alberto Aquili, Antonio Gigante

**Affiliations:** 1 Faculty of Medicine, Polytechnic University of Marche, Via Tronto 10/A, Ancona IT-60126, Italy; 2 Department of Industrial Engineering & Mathematical Sciences, Faculty of Engineering, Polytechnic University of Marche, Via Brecce Bianche, Ancona IT-60131, Italy; elmehtedi@univpm.it; 3 Clinical Orthopaedics, Department of Clinical and Molecular Sciences, Faculty of Medicine, Polytechnic University of Marche, Via Tronto 10/A, Ancona IT-60126, Italy; bottegonicarlo@gmail.com (C.B.); a.aquili@univpm.it (A.A.); a.gigante@univpm.it (A.G.)

**Keywords:** chitosan, genipin, tissue engineering, biomedical uses, biochemical properties

## Abstract

The present review article intends to direct attention to the technological advances made since 2009 in the area of genipin-crosslinked chitosan (GEN-chitosan) hydrogels. After a concise introduction on the well recognized characteristics of medical grade chitosan and food grade genipin, the properties of GEN-chitosan obtained with a safe, spontaneous and irreversible chemical reaction, and the quality assessment of the gels are reviewed. The antibacterial activity of GEN-chitosan has been well assessed in the treatment of gastric infections supported by *Helicobacter pylori*. Therapies based on chitosan alginate crosslinked with genipin include stem cell transplantation, and development of contraction free biomaterials suitable for cartilage engineering. Collagen, gelatin and other proteins have been associated to said hydrogels in view of the regeneration of the cartilage. Viability and proliferation of fibroblasts were impressively enhanced upon addition of poly-l-lysine. The modulation of the osteocytes has been achieved in various ways by applying advanced technologies such as 3D-plotting and electrospinning of biomimetic scaffolds, with optional addition of nano hydroxyapatite to the formulations. A wealth of biotechnological advances and know-how has permitted reaching outstanding results in crucial areas such as cranio-facial surgery, orthopedics and dentistry. It is mandatory to use scaffolds fully characterized in terms of porosity, pore size, swelling, wettability, compressive strength, and degree of acetylation, if the osteogenic differentiation of human mesenchymal stem cells is sought: in fact, the novel characteristics imparted by GEN-chitosan must be simultaneously of physico-chemical and cytological nature. Owing to their high standard, the scientific publications dated 2010–2015 have met the expectations of an interdisciplinary audience.

## 1. Introduction and Scope

The most important applications of genipin in conjunction with chitosan are the preparation of elastic cartilage substitutes, the manufacture of carriers for the controlled release of drugs, the encapsulation of biological products and living cells, the biofabrication of tissues such as muscle and arterial walls, and the dressing of wounds in animals and humans. Genipin has definitely replaced glutaraldehyde and other crosslinkers mainly owing to the expanded biochemical significance of the genipin-crosslinked hydrogels (GEN-chitosan), but also owing to the advantages of stability, biocompatibility, well defined chemistry and general safety of the products whose manipulation, handling and quality assessment are currently done with advanced techniques and clearly defined protocols that guarantee absence of cytotoxicity.

### 1.1. Characteristic Properties of Genipin

The first review article on genipin was published in 2009 [1], but two early papers [2,3] on the isolation and structure of genipin deserve to be cited here because they are valid examples of exhaustive research and scientific soundness obtained with advanced equipment. Working in the early 1950s with Syntex S.A. in Mexico City, Carl Djerassi first synthesized 19-nor-17α-ethynyltestosterone (norethisterone). This steroid, derived from inedible yams of a wild plant *Dioscorea*, proved to be the most effective orally administered progestational agent discovered at that time. This was the start of a very fortunate research program that led to hundreds and hundreds of journal articles and patents. Syntex could boast of possessing the most advanced equipment such infrared and NMR spectrometers, at a time when neither the pharmaceutical industries, as Djerassi wrote, “nor my Alma Mater, the University of Wisconsin, had such equipment which proved to be enormously useful for steroid research” [4]. The work done paved the way to the first synthesis of a steroid contraceptive in 1953, “the Pill” that changed the habits of mankind [4]. In the frame of said research program, several other plants were investigated and several extracts were described scientifically with avalanches of data, thus starting the evolution of the empirical medicaments of the traditional medicine into scientifically assessed plant extracts, as it was the case of *Genipa americana* and *Gardenia jasminoides* Ellis that yielded commercial genipin. A more recent example is the food supplement from *Serenoa repens* (Permixon™ Pierre Fabre, Giem, France). Thus genipin is a part of the cultural legacy from Carl Djerassi.

Because it is recognized that genipin, rather than geniposide, is the main compound that exerts pharmacological activities [5], there is interest in its isolation and purification for use in therapy and in the manufacture of food commodities [6]. Genipin is choleretic; anti-depressant; antidiabetic; anticancer; antithrombotic; anti-inflammatory; antibacterial; gastro-, hepato-, and neuro-protective [7]; it prevents lipid peroxidation; and it protects the hippocampal neurons against the Alzheimer’s amyloid beta protein [8].

The biochemical significance of genipin emerges in fact from a number of research projects in the areas of the therapies of vascular diseases, diabetes, hepatic dysfunctions, as well as biofabrication, dentistry, ophthalmology, wound healing and regeneration of nerve, tendon and other tissues, just to mention a few [9,10,11,12,13,14,15,16,17,18,19,20].

The main specifications of genipin (CAS 6902-77.8) are the following: white crystalline powder soluble in water, methanol, ethanol and acetone; chemical formula C_11_H_14_O_5_; molar mass 226.226 g/mol; melting point 120–121 °C; UV (CH_3_OH) λ_max_ 240 nm.

Although a minor molar ratio of genipin to chitosan is necessary for crosslinking the latter or other aminated polymers, genipin is expensive because during its preparation a large quantity is wasted owing to homopolymerization. Therefore *Fusarium solani* was screened as an efficient source of β-glucosidase for genipin preparation from geniposide by extraction with a 10-L ethyl acetate-water biphasic system. HPLC data indicated that immediately after hydrolysis genipin was extracted from the aqueous phase into ethyl acetate thus escaping homopolymerization that would have been unavoidable in the aqueous phase. With *Fusarium solani* ACCC 36223, genipin in the ethyl acetate phase was 15.7 g/L, corresponding to yields of 0.65 g·L^−1^·h^−1^. Efficient substrate conversion and side reactions elimination were the key aspects of the advances made; moreover genipin was easily purified via the sole recrystallization. These most recent conceptual and technical approaches will certainly permit a more convenient production at lower price [21]. The available methods for recovery of genipin and geniposide were described, as well as the methods for genipin and geniposide identification and quantification based on instrumental analyses. Analytical methods for genipin were implemented in view of effective recovery protocols [22,23,24,25,26,27,28,29,30,31,32,33,34,35,36,37].

### 1.2. Characteristic Properties of Chitosans

Chitins and chitosans of various origins along with some of their derivatives are today protagonists in the scenario of wound healing, tissue engineering, gene therapy, and other advanced biomedical areas, owing to their unique properties. Basic information on these polysaccharides, relevant to the title topic, can be found in books and review articles [38,39,40,41,42,43,44,45,46,47,48,49].

Being biocompatible, non-toxic, stable, sterilizable and biodegradable, chitosan exhibits most appreciated properties that enhance its versatility in the biomedical and biotechnological fields, such as immunostimulation, activation of macrophages, mucoadhesion, antimicrobial activity, and well assessed chemistry [50]. Moreover, chitosan can also be prepared in a variety of forms, namely hydrogels and xerogels, powders, beads, films, tablets, capsules, microspheres, microparticles, nanofibrils, textile fibers, and inorganic composites. Chitosan is today a protagonist in advanced fields, for example it is a high performing non-viral vector for DNA and gene delivery.

### 1.3. Genipin-Crosslinked Chitosan Hydrogels

**Figure 1:**
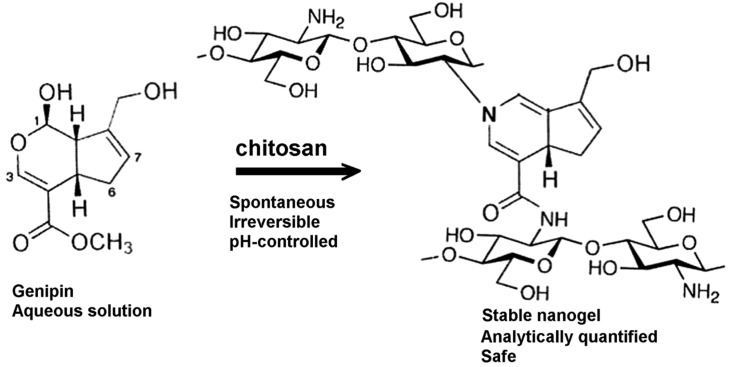
Genipin crosslinks chitosan spontaneously at a quite small molar ratio. On the right, two chitosan chains (represented by their structural units) react covalently with one mole of genipin to yield two newly formed chemical functions, namely the monosubstituted amide and the tertiary amine.

Genipin reacts promptly with chitosan, as well as with proteins or amines in general [51], as a bi-functional crosslinking compound, thus producing blue-colored fluorescent hydrogels. The reaction between chitosan and genipin is well understood for a variety of experimental conditions and yields composites and complexes with no cytotoxicity for human and animal cells (Figure 1).

**Figure 2:**
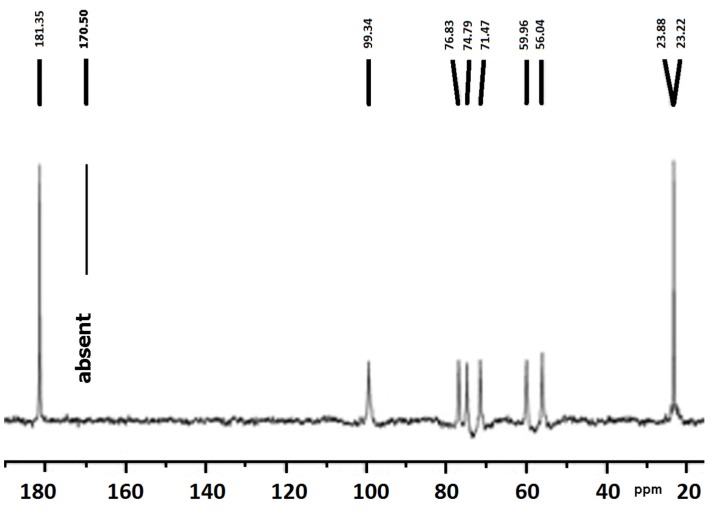
^13^C NMR spectrum of chitosan film crosslinked with genipin 0.10%. At 23.0 ppm the resonance signal of alkyl groups in the crosslinked chitosan was attributed to the chitosan + genipin linkage. The signal at 170.5 ppm, assigned to the ester group of plain genipin, disappeared as a consequence of the reaction, thus the resonance at 181.3 ppm is assigned to the amide generated by the reaction between the amino group of chitosan and the ester group of genipin.

Chitosan nanoparticles crosslinked with genipin were prepared by reverse microemulsion that allowed obtaining highly monodisperse nanogels. Whilst ^13^C·NMR provides evidence of the reaction as shown in Figure 2, the incorporation of genipin into chitosan was also confirmed and quantitatively evaluated by ^1^H·NMR [52,53]. The hydrodynamic diameter of the genipin-chitosan nanogels ranged from 270 to 390 nm and no difference was found when the crosslinking degree was varied. The hydrodynamic diameters of the nanoparticles increased slightly at acidic pH. TEM data indicated that the nanoparticles had average diameters of from 3 to 20 nm and that they are spherical, have nearly uniform particle size distribution, and are not affected by particle agglomeration; these being interesting qualities for drug delivery. The progressive protonation of the amino groups as pH decreases was confirmed by measuring the electrokinetic potential of the nanogels. The variation of water solubility of chitosan due to the crosslinking with genipin is a compromise between the decrease of crystallinity and the elastic force within the generated network. There was an insignificant variation of the average hydrodynamic diameter of the nanoparticles with pH, but a large progressive variation of zeta potential (from +30 to −7 mV) in the pH interval 4–9, indicative of the fact that these hydrogels are pH-sensitive [53].

Biodegradable polymers such as chitosan need to be crosslinked in order to modulate their general properties and to last long enough for delivering drugs over a desired period of time. Certain chemicals have been used for crosslinking chitosan such as glutaraldehyde, tripolyphosphate, ethylene glycol, diglycidyl ether and diisocyanate. However, the synthetic crosslinking reagents are all more or less cytotoxic and may impair the biocompatibility of a chitosan delivery system. Hence, efforts were made to provide crosslinking reagents that have low cytotoxicity and that form stable and biocompatible crosslinked products, for example tyrosinase was used to mediate quinone tanning of chitosans [54].

Chitosan can be used as a scaffold for tissue regeneration in porous or film form. However, as a porous scaffold it exhibits mechanical weakness: for example, when mouse fibroblasts are cultured on a porous chitosan scaffold, the narrow site of attachment and general weakness drastically depress the adhesion, and the cells tend to become round thus losing their prerogatives. On the other hand, when the cells are anchored to a surface endowed with stiffness, the cellular growth and differentiation rates are better, migration and aggregation become evident, and the cellular shapes favored by the support are those associated with proliferation, differentiation, and apoptosis.

A number of research teams are interested in using genipin to obtain stable and biocompatible chitosan hydrogels. Yao *et al.* indicated that the fibroblasts adhering to the GEN-chitosan scaffolds were 2.29 times more numerous compared to the fibroblasts on the pristine scaffold surface, the characteristic modulus of a genipin-crosslinked chitosan surface, ≈2.3 GPa, being nearly the double of the control [55]. A genipin crosslinked scaffold retains its own chemical composition while having significantly larger Young’s modulus and hardness. Thus, the mechanical properties of a porous chitosan scaffold in film form are enhanced by genipin. In turn the enhanced general properties induce cell adhesion and proliferation in the modified porous scaffold. Interestingly, the pore size and mechanical properties of chitosan can be tuned for specific tissue regeneration.

Moreover, survival and proliferation of L929 fibroblasts were up-regulated after crosslinking with genipin, especially 0.5% genipin solutions. Analogous data were presented by Bao *et al.* for carboxymethylchitosan crosslinked with genipin in an article devoted to the mechanical properties of that class of hydrogels and their biocompatibility [56].

GEN-Chitosan hydrogels were prepared by incubation of solutions containing mixtures of genipin and chitosan in different ratios. They turned dark blue and became opaque, owing to exaggerated quantity of genipin. Upon lyophilization they yielded macroporous sponge-like scaffolds [57]. The *in vitro* cytocompatibility of hydrogels was demonstrated with L929 fibroblasts by the MTT method, in agreement with other authors [58]. The macroporous structure of the chitosan hydrogels could be tailored so that they enhanced their storage modulus, and also altered their hydrophilicity and swelling properties. The crosslinked hydrogels did not induce cytotoxic effects. Flow cytometry showed that fibroblasts possessed good viability on the surface of crosslinked gels (88.4%–90.9%) close to that on blank plates (93.7%) and chitosan films (92.8%). There was no quantitative difference in apoptotic or dead cells, thus crosslinking had little influence on viability, but the stiffness was the most important parameter influencing cell growth and made it possible to switch the cells either toward round or spreading shapes upon modulation of the hydrogel stiffness.

**Figure 3:**
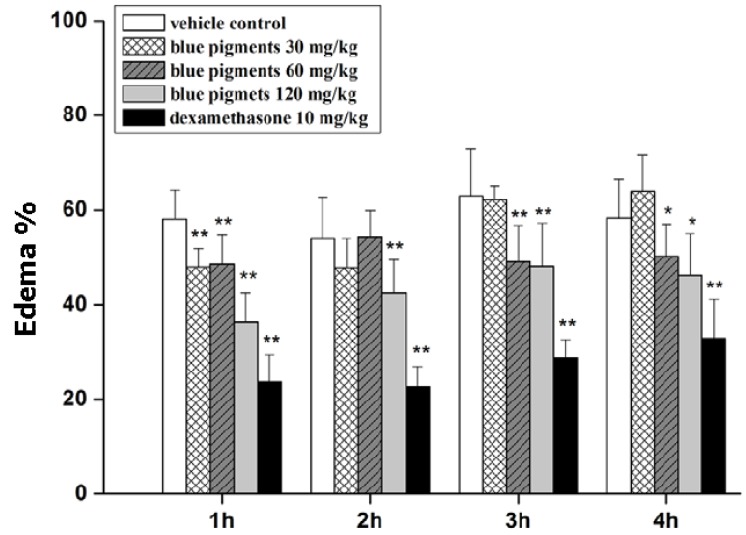
Anti-inflammatory effect of genipin + glycine blue pigment on edema in mice. Maximum edema depression was observed 1 h after edema induction. Notably, treatment with blue pigments at 120 mg/kg reduced edema by *ca.* 22% (from *ca.* 60% to 38%) at 1 h, whereas the positive control, dexamethasone (10 mg/kg) depressed the edema by *ca.* 35% at 1 h. The data are indicative of the safety of genipin which alleviates inflammation by exerting biochemical actions favorable to the organism. Reproduced from [33].

Safety of use was amply confirmed, thus chitosan composites have been taken into consideration in view of the production of biomaterials with desirable physicochemical and biological properties for tissue engineering. It is worth emphasizing that the safety of genipin has been demonstrated by a number of approaches, for example although the blue pigments derived from genipin and aminoacids have been used as value-added colorants for foods over the last 20 years in Eastern Asia, their biochemical significance has been explored as recently as in 2012 by Wang, Q.S. *et al.* who demonstrated that blue pigments did not only inhibit iNOS and COX-2 gene expression induced by LPS and subsequent production of NO and PGE_2_, but reduced the production of cytokines (TNF-α, IL-6) induced by LPS in macrophages by the inhibition of signaling cascades leading to the activation of NF-κB [33]. Therefore, the results of recent studies provide strong scientific evidence for blue pigments to be developed as nutraceuticals for prevention and treatment of chronic inflammatory diseases. Nitric oxide is recognized as a mediator and regulator of inflammatory responses being produced in high amounts by iNOS in activated inflammatory cells. Blue pigments were found to inhibit LPS-induced NO production (Figure 3). Also the mRNA expression of iNOS was decreased by blue pigments, confirming the inhibitory effect of blue pigments on the NO production. That work also showed that blue pigments inhibited the expression of iNOS mRNA in LPS-stimulated macrophages. The effect of blue pigments on LPS-induced iNOS expression might result from the transcriptional inhibition of the iNOS gene. Further, the anti-inflammatory effect of blue pigments might be attributed to their inhibitory effect on PGE_2_ production through blocking COX-2 gene and protein expression. Therefore, besides being safe, genipin is also beneficial owing to its positive action when present in functional foods.

### 1.4. Scope

The scope of this review article is therefore the evaluation of recent data on the title crosslinked hydrogels and scaffolds for (i) the description and appreciation of the experimental advances foreseen in the earlier review article by Muzzarelli [1], and actually performed in the most recent years; (ii) efficacy of said compounds in the upgrading of the chemical and biochemical characteristic properties and in their capacity to modulate the behavior of cells and stem cells *in vitro* and *in vivo*; (iii) treatments for the regeneration of the joint cartilage; and (iv) treatment and materials for enhanced osteogenesis and for the regeneration of bone, optionally including inorganic composites. Aspects related to sources of the raw materials, analytical chemistry, drug delivery, and economics are also considered. Understanding the synergy of the two ingredients of this class of composites in providing safety of use and efficacy in the pre-clinical trials is a further object of this work.

## 2. Therapies Based on the Genipin-Crosslinked Chitosan Alginate Complex

The encapsulation technology permits long-term delivery of desired therapeutic products to certain parts of the body without the use of immuno-suppressant drugs. In the study by Nayak *et al.* microcapsules composed of sericin and alginate micro bead as inner core with the outer chitosan shell were prepared for therapeutic applications [59]. The sericin-alginate micro beads were prepared via ionotropic gelation under high applied voltage and were coated with chitosan and crosslinked with genipin, their size (300–800 µm) depending on flow rate and applied voltage. Alamar Blue assay and confocal microscopy showed high cell viability and uniform cell distribution within the sericin-alginate-chitosan microcapsules indicative of the favorable internal microenvironment for the cells. In fact glucose consumption, urea secretion rate and intracellular albumin content increased in the microcapsules. The genipin crosslinked chitosan provided a fluorescent coating around the capsules, that appear light blue in the visible light. The coating is mandatory for the chemical stability of the capsules, particularly when dealing with *in vivo* delivery for therapeutic purposes: in fact, encapsulated hepatocytes generated enriched populations of metabolically and functionally active cells of therapeutic usefulness in acute liver failure.

Covalent crosslinking with genipin of chitosan alginate microcapsules provides significant enhancement of the microcapsule strength and resistance while maintaining the permeability. Aldana *et al.* reported the compatibility of genipin with other polymers such as polyvinylpyrrolidone and its suitability in making the soft, tough material for controlled drug release [60]. Moisture absorption can be modulated even in polyamide 6,6 fabrics when the surface is functionalized with the aid of GEN-chitosan hydrogels [61].

Scaffolds of chitosan-coated alginate were fabricated in a layer-by-layer fashion by Colosi *et al.* for drug delivery. A dispensing system based on two coaxial needles delivered alginate and calcium chloride solutions yielding alginate fibers according to designed patterns. Coating of the alginate fiber with chitosan and subsequent crosslinking with genipin assured the endurance of the scaffold. The crosslinking imparted to the scaffold a hierarchical chemical structure. Typical hepatic functions such as albumin and urea secretion and induction of CYP3A4 enzyme activity following drug administration were quite good [62].

Further chemical characteristics of the GEN-chitosan alginate combinations were reported by a number of authors: Silva *et al.* [63,64] further assessed the advantages of the LbL technique to generate functional biomimetic surfaces with tuned mechanical and chemical properties, and for the preparation of nanostructured multilayers tubes combining LbL and template leaching. Those works demonstrate the versatility and feasibility of LbL assembly to generate nanostructured devices including freestanding membranes with tunable permeability, besides mechanical and biological properties, by acting on the molar ratios of each polysaccharide and genipin.

Microcapsules with a calcium alginate core and a genipin-crosslinked chitosan alginate coating were prepared by Ranganath *et al.* [65] with good control over size, membrane thickness and density. Importantly, the authors interrelated membrane thickness, chitosan + alginate reaction rate constant, and diffusion coefficient. The large immunoglobulin and carbonic anhydrase were found to diffuse promptly. Compared to other microcapsules, the genipin treated microcapsules exhibited improved permselectivity of small nutrient compounds and proteins, while excluding antibodies.

## 3. Stem Cells in Regenerative Medicine

Many studies have indicated that human adipose-derived stem cells can easily be obtained from liposuction waste or arthroscopy, and maintained in a stable undifferentiated state during *in vitro* expansion [66]. Although ASC can be induced toward a chondrogenic phenotype with growth factors, that would make them suitable for cartilage regeneration, the use of exogenous growth factors may be impractical for clinical use owing to economic or regulatory issues. Instead, a bioactive scaffold exhibiting appropriate environmental signals may provide an alternative approach for inducing ASC chondrogenesis.

Stem cell transplantation has enormous potential in regenerative medicine [67,68]. Microencapsulation of stem cells is an efficient procedure for the preservation of viability and biochemical properties especially for the therapy of heart diseases. Paul *et al.* reported the use of microcapsules made of GEN-chitosan alginate for the delivery of human adipose stem cells (hASC) with the aim to increase the implant retention in the infarcted myocardium for maximum therapeutic benefit [69]. Under hypoxic conditions *in vitro*, the microencapsulated cells overexpressed higher amount of biologically active vascular endothelial growth factor (VEGF), thus the *in vivo* potential was investigated by using immunocompetent rats after induction of myocardial infarction. For this, rat groups received either empty control microcapsules, or 1.5 × 10^6^ free hASC, or 1.5 × 10^6^ microencapsulated hASC. Results showed significant retention (3.5-fold higher) of microencapsulated hASCs compared to free hASCs 10 weeks after transplantation. Microencapsulated hASC led to attenuated infarct size compared to the free hASC group and the empty microcapsule group, besides enhanced vasculogenesis and improved cardiac function. Therefore, the GEN-chitosan alginate microcapsules are deemed to be a valid aid for the significant improvement of the cardiac functions.

Porous cartilage-derived matrix (CDM) from porcine articular cartilage induced *in vitro* chondrogenic differentiation of adult human stem cells or chondrocytes without exogenous growth factors. Cheng *et al.* 2011 investigated CDM scaffolds crosslinked with genipin, seeded with ASC, and then cultured for four weeks [70]. By using a 0.05% genipin solution, a crosslinking degree of 50% was achieved (involving *ca.* one-half of the available lysine or hydroxylysine units in the cross linkage), and the ASC-seeded constructs exhibited no significant contraction during the culture. Moreover, the expression of cartilage-specific genes, the accumulation of cartilage-related macromolecules and the development of mechanical properties were comparable to the original CDM, thus making the cartilage-derived matrix crosslinked with genipin a contraction-free biomaterial suitable for cartilage tissue engineering [71]. Contraction of engineered cartilage *in vivo* invariably creates a gap between the construct and the nearby native cartilage. The fact that integration of engineered scaffolds with surrounding native tissue is crucial for both immediate functionality and long-term performance of the tissue enables one to appreciate the important contribution of genipin in solving this issue particularly crucial for articular cartilage repair because the surrounding native cartilage has scarce regeneration potential.

## 4. Genipin-Crosslinked Chitosan in Gastric Infections

Chitosan microspheres have been explored for pharmaceutical applications as drug delivery hydrogels in particular for the treatment of *Helicobacter pylori* gastric infection, owing to their mucoadhesive capacity. *H. pylori* is an important human pathogen that recognizes specific carbohydrate receptors, such as the fucose receptor, and produces the vacuolating cytotoxin, which induces inflammatory responses and modulates the cell junction integrity of the gastric epithelium. Nogueira *et al.* [72] proposed a different application of chitosan microspheres that capture and remove those bacteria from infected patients, taking advantage of their adhesive capacity for mucins and bacteria: they studied the effect of genipin on stability, size, charge and mucoadhesion of chitosan microspheres in acidic media. Chitosan microspheres (*ca.* 170 μm) were produced by ionotropic gelation and subsequently covalently crosslinked with genipin to various extents. Both the zeta potential and the swelling capacity of chitosan microspheres decreased with increasing crosslinking. When immersed in simulated gastric fluid with pepsin for seven days, the microspheres crosslinked with 10 mM genipin for 1 h presented an adequate balance between capacity to bind mucins, and free amino groups required for maintaining chitosan stability in acidic environment, and had gastric retention time *ca.* 2 h *in vivo*; they did not dissolve but simply doubled their size to *ca.* 345 μm. Although they maintained their *in vitro* mucoadhesion to soluble gastric mucins at pH 3.6 and 6.5 and presented an *in vivo* retention time of *ca.* 2 h in the stomach of mice, they were unable to lead to satisfactory results owing to the presence of pepsin [72]. Delmar *et al.* found that although the reaction between chitosan and genipin is apparently slow and might require up to four days for completion, the alteration of the pH within the small range of 4.00–5.50 dramatically affects the reaction, yielding hydrogels differing in appearance and properties [73]. The ability to manipulate the hydrogel properties, while adjusting the conditions slightly, provides a powerful and useful tool when designing chitosan hydrogels. Furthermore, the dependence of the properties on tiny pH modifications is crucial when reproducible and reliable results are sought.

On the other hand, Lin, Y.H. *et al.* [74] combined fucose-conjugated chitosan with genipin in genipin-crosslinked fucose-chitosan/heparin nanoparticles to encapsulate amoxicillin and straightforwardly make contact with the bacterium on the gastric epithelium. The nanoparticles effectively reduced drug release to gastric acids, and then released amoxicillin to inhibit *H. pylori* growth, and reduced disruption of the cell junction protein in the infected areas. Thus, with amoxicillin-loaded nanoparticles, a more complete *H. pylori* clearance effect was observed, and the *H. pylori* associated gastric inflammation in an infected animal model was definitely reduced. Thakur *et al.* also made use of highly stable GEN-chitosan beads in simulated gastric and intestinal fluids for the release of amoxicillin [75].

### Further Aspects of Enhanced Antibacterial Efficacy

The antibacterial efficacy of GEN-chitosan has been well assessed by Wang R *et al.* who mixed the antifouling polymer poly(sulfobetaine methacrylate) and the bactericidal *N*-[(2-hydroxy-3-trimethylammonium) propyl] chitosan, in one coating onto a silicone surface, by using genipin [76]. Yu, S.H. *et al.* developed fucoidan-shelled chitosan beads with the purpose of oral delivery of berberine to inhibit the growth of bacteria [20]. Furthermore, a nanoparticles + beads complex was developed by incorporation of berberine-loaded chitosan + fucoidan nanoparticles in the fucoidan-shelled chitosan beads. It served as a drug carrier to delay the berberine release in simulated gastric fluid, with lag time of 2 h, and it effectively inhibited the growth of common clinical pathogens.

Drug administration via the oral mucosa is an attractive strategy owing to good patient compliance, prolonged localized drug effect, and avoidance of gastrointestinal drug metabolism and first-pass elimination. Oral drug delivery systems need to maintain an intimate contact with the mucosa lining in the wet conditions of the oral cavity for long enough to allow drug release and absorption. Chitosan and its derivatives have been examined for this purpose. In particular, the genipin treated carboxymethyl–hexanoyl chitosan, an amphiphilic chitosan derivative with quite good swelling ability, cytocompatibility and water solubility, was studied under physiological conditions [15]. Inspired by the wet adhesion of marine mussel adhesive protein, Xu, J.K. *et al.* [77] developed an oral drug delivery system using a catechol-chitosan hydrogel. The catechol functional groups were covalently linked to chitosan, and the resulting modified chitosan was crosslinked with genipin. Catechol groups significantly enhanced mucoadhesion *in vitro* when in contact with porcine mucosal membrane up to 6 h, whereas the chitosan hydrogels lost contact after 1.5 h. The new hydrogel systems sustained the release of lidocaine for about 3 h. *In vivo*, buccal patches adhered to rabbit buccal mucosa, thus lidocaine was monitored easily in the rabbit serum owing to the intimate contact provided by the highly mucoadhesive catechol-GEN-chitosan [77].

## 5. Genipin-Crosslinked Collagen/Gelatin for the Regeneration of the Cartilage

Collagen and gelatin have been treated with genipin in a number of instances with the intention to involve them in the treatment of cartilage: in their review Elzoghby *et al.* reported that the mechanism of crosslinking of proteins by genipin involves the free amino groups of lysine in the protein [78]. Recent advances on the regeneration of cartilage have been reviewed by Muzzarelli *et al.* [79] and Bottegoni *et al.* [80]. Because a crosslinker is necessary to improve and optimize mechanical strength, porosity and degradability of single biopolymers and their composites, Bi, L. *et al.* crosslinked chitosan + collagen scaffolds by using genipin [81]: the compressive strength was directly dependent on the genipin concentration in the interval 0.1% to 1.0% and on the crosslinking time. The pore size, degradation rate and swelling ratio changed significantly with different crosslinking conditions. For a similar genipin crosslinked chitosan + collagen material, Yan, L.P. *et al.* found that rabbit chondrocytes adhered well to the surface of the scaffolds and reached confluence, thus they suggested that the genipin crosslinked chitosan plus collagen may be a promising formulation for articular cartilage scaffolding [82].

Genipin-crosslinked recombinant human gelatin (preferred owing to its homogeneity in molecular weight and precisely defined properties) was efficiently internalized in the cells without inducing cytotoxicity. Genipin was also used to stabilize the structure of gelatin–dextran micelles encapsulating tea polyphenol to avoid disintegration after dilution: the crosslinked micelles were stable with no size change. Kuo *et al.* preferred bovine pituitary extract for study of the formation of neocartilage in chitosan/gelatin scaffolds, and cultured bovine knee chondrocytes in it over 28 days; collagen-II was synthesized in the constructs, thus demonstrating the chondrocytic phenotype of proliferated chondrocytes [83]. Yin *et al.* blended chitosan plus polylactide with collagen-II to fabricate layered composites potentially applicable in cartilage repair [84]. The manufacture of marine collagen porous structures crosslinked with genipin under high pressure CO_2_ was investigated by Fernandes-Silva *et al.*: shark skin collagen was used to prepare prescaffolds by freeze-drying. Under dense CO_2_ atmosphere, crosslinking of collagen with genipin was protracted for 16 h [85].

Modulation of the proliferation and matrix synthesis of chondrocytes by dynamic compression on genipin-crosslinked chitosan plus collagen scaffolds was also observed [86]. Dynamic compression is an important physical stimulus for the physiology of chondrocytes and engineering of the articular cartilage. Rabbit chondrocytes were seeded in genipin-crosslinked chitosan plus collagen and then cultured for three days prior to two weeks of cyclic compression of 40% strain, 0.1 Hz, and 30 min/day. The cell proliferation and the total GAG deposition was directly dependent on genipin quantity and dynamic compression.

While fully biocompatible gelatin microspheres for intra-articular drug delivery were prepared by Kawadkar *et al.* [87], emulsion-crosslinking was used by Kawadkar and Chauhan [88] to prepare chitosan microspheres with various concentrations of genipin and drug-to-polymer ratios for intra-articular delivery of flurbiprofen. The mean particle size was in the range 5.18–9.74 μm with drug entrapment up to 81%. The optimized microspheres were able to release the drug for more than 108 h. The biocompatibility of the microspheres in the rat knee joints was confirmed by histopathology. Pharmacokinetic data pointed out the extended release of flurbiprofen from microspheres in comparison with solution, so that GEN-chitosan qualified as an injectable drug vehicle. According to the *in vivo* data, the microspheres made of chitosan and genipin are safe for the synovia and maintain the drug concentration within the arthritic knee joint. In fact, Sarem *et al.* explained how genipin helps chitosan with gelatin scaffolds act as replacements of load-bearing soft tissues and concluded that the 1% genipin-crosslinked chitosan 40 with gelatin 60 scaffolds, prepared at room temperature for 24 h was a promising replacement of missing segments of load-bearing soft tissues. Owing to the hydrogel characteristics of said biopolymers, a significant amount of fluid can be retained in their structure. Hence, they can produce high compressive modulus comparable to native load-bearing soft tissues: these materials can be used for treatment or repair of articular cartilage and meniscus. This was attributed to the formation of polyelectrolyte complexes via ionic interactions between the amino groups of chitosan and the anionic groups in gelatin. Finally the presence of genipin depressed the depolymerization of chitosan by lysozyme, while still permitting an adequate degradation and ingrowth of newly formed tissues, *i.e.*, remodeling of tissues under loadbearing conditions [89].

The genipin-crosslinked chitosan + gelatin scaffolds containing bovine pituitary extract are quite effective in the regeneration of neocartilage. The histological and immunochemical staining showed chondrogenesis in the culture of bovine knee condrocytes using said scaffolds in a medium containing bovine pituitary extract. In addition, collagen-II was synthesized in the constructs, demonstrating the chondrocytic phenotype of proliferated bovine knee chondrocytes in said scaffolds over 28-day culture. In practice, the addition of the extract to the culture medium accelerated the regeneration of the articular cartilage [83].

**Figure 4:**
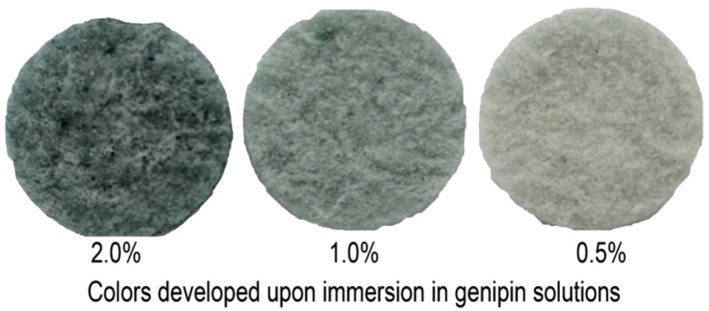
Chitosan-protein scaffolds crosslinked with genipin at various concentrations, under identical conditions. The intensity of the blue color is an indication of the extent of crosslinking.

Scaffolds made of chitosan, collagen and gelatin were prepared with the aid of carbon dioxide saturated solutions [90], the chitosan dissolution in carbonic acid being no longer a laboratory curiosity. Chitosan was dissolved upon saturation of an aqueous colloidal chitosan suspension with gaseous CO_2_ under mild conditions: atmospheric pressure and room temperature. As CO_2_ dissolves in water, the pH decreases owing to formation of carbonic acid. This is a fine demonstration that commonly used inorganic and organic acids are no longer indispensable for the dissolution of chitosan. Moreover, this approach simplifies and optimizes the preparation of wound dressing materials, where the presence of undesirable and cytotoxic counter ions such as acetate is avoided. The use of CO_2_ for chitosan dissolution made the scaffold preparation more reproducible and economically sustainable. Porosity data are in Table 1; the values of other parameters were: dissolution degree (30%), lysozyme-induced degradation (5% after 168 h), good antioxidant properties, and especially absence of cytotoxicity against mouse NIH 3T3 fibroblasts, the viability being at the level of the control. The fibroblasts grew uniformly in the pores of the chitosan-protein structure owing to optimal swelling of the scaffold and even distribution of collagen, to which cells have high affinity. When the chitosan + protein scaffolds are treated with genipin, the color intensity reveals the extent of the crosslinking, as shown in Figure 4.

**Table 1:** Average cross-sectional areas of the pores and porosity of the chitosan-protein scaffolds crosslinked with different concentrations of genipin. The reaction time and temperature are not specified and depended on the protocol adopted.

Genipin Concentration (%, *w*/*w*)	Cross-Sectional Area of the Pores (μm^2^)	Porosity (%)
0.0	187.9 ± 101.0	25.75 ± 1.47
0.5	274.6 ± 123.8	33.13 ± 1.30
1.0	533.9 ± 259.8	39.95 ± 1.25
2.0	1066.4 ± 396.7	44.75 ± 1.50

Data from [90], and set in novel tabular presentation.

### 5.1. Fibrin, Poly-l-Lysine, Heparin, Hyaluronan

Fibrin is another biopolymer studied in conjunction with chitosan and genipin, in order to develop biocompatible microspheres. Human chondrocytes cultured on the composite substrate were viable during the culture period (28 days): at the end the composite substrate showed 41% more collagen-II and 13% higher production of sulfated glycosaminoglycans with respect to the amounts found at 14 days. The de-differentiated chondrocytes cultured in monolayer on the composite could re-acquire characteristics of differentiated cells without using three-dimensional substrates or chondrogenic media [91].

Nihn *et al.* established a quantitative framework for controlled release systems in order to deliver genipin into protein-based hydrogels. Covalent coupling between genipin and primary amines in fibrin gels obeys second-order kinetics in genipin concentration with an effective activation energy of −71.9 ± 3.2 kJ·mol^−1^. Genipin-crosslinked fibrin clots are resistant to fibrinolytic degradation as measured by rheology. Interestingly, active genipin can be delivered from poly(d,l-lactide-co-glycolide) matrices to gels at rates that are comparable to the characteristic rate of incorporation in fibrin networks. Poly-l-lysine, the homopolymer of the essential aminoacid Lys, has been used in other works as a standard tissue culture coating to promote cellular adhesion. Films manufactured after blending it with chitosan enhance cellular attachment to chitosan. Genipin improves and maintains the stability of chitosan + poly-l-lysine blends [92]. In the article by Mekhail *et al.*, the viability of fibroblasts was enhanced more than six-fold after treating with genipin the gels containing nearly equal weight of the two polymers; the proliferation was enhanced up to five folds. Fibroblast viability was significantly enhanced after crosslinking the 60:40 and 50:50 gels; whilst it was not on 100 and 80:20 gels [93]. This is in agreement with data by other groups that reported that genipin improves the biocompatibility of various chitosan formulations [94,95,96]. Heparin was covalently crosslinked to the chitosan scaffolds by using genipin, which bound fibroblast growth factor-2 (FGF-2) while preserving its biological activity. At 1 µg/mL approximately 80% of the FGF-2 bound to the scaffold that showed good cytocompatibility and therefore could be used for the delivery of neural stem cells and growth factors for central nervous system repair [97]. Likewise, carrageenan and carboxymethylcellulose have been studied in conjunction with genipin [98,99].

Finally, a few words on hyaluronan, a polysaccharide possessing aspects of chemical similarity to chitosan. Jalani *et al.* [100] reported a method to produce tough, thermogelling, safe, injectable hydrogels made of chitosan and hyaluronan co-crosslinked with β-glycerophophate and genipin. The highly homogeneous gels form within half an hour, *i.e.*, faster than gels crosslinked with either genipin or β-glycerophosphate. The shear strength of co-crosslinked hydrogels was 3.5 kPa, higher than for any chitosan-based gel reported. Chondrocytes and nucleus pulposus cells thrive inside the gels and produce large amounts of collagen-II. The gelation took place *in vivo* within a short time after injection in rats and remained well localized for more than one week while the rats were healthy and active [100].

Swelling and degradation of the chitosan + hyaluronan complex could be controlled with the aid of genipin. Optimization of said parameters is important because cells need to adhere first to the scaffold and then proliferate. BMP-2 was immobilized in the complex by electrostatic attraction. Thus, high loading and sustained BMP-2 release were achieved. Reverse transcriptase PCR indicated that released BMP-2 facilitated osteogenesis at all stages, this being a key factor for bone regeneration [101].

### 5.2. Genipin Treatment of Tendon Cells and Matrix

Fessel *et al.* studied the effects of genipin treatment on tendon cells and their matrix, with a view to *in vivo* application to the repair of partial tendon tears [102]. They observed that post-treatment cell survival may be adequate to eventually repopulate and stabilize the tissue. Superficial blue pigmentation that qualitatively indicates genipin was documented. Inherent sample fluorescence (λ_ex_ 510–560 nm; λ_em_ 590 nm) was measured with the aid of fluorescence microscopy: uniform fluorescence throughout the cut sections indicated homogeneous crosslinking. According to a model the cell viability in tendon explants was concentration dependent, but cell survival was not time dependent. The model predicted that *ca.* 50% of cells would remain viable at genipin concentrations of 6.2 mM applied for 72 h, with decreasing cell viability after prolonged incubation. While many cells remained alive with genipin 5 mM or less for the time spans studied, effects on cell metabolism occurred at lower concentrations. The model predicted a 50% drop in metabolic activity at 0.4 mM after 72 h, this being consistent with reduced cell motility at similar concentrations. Although reduced collagen-I expression was also consistent with reduced metabolic activity, apoptosis markers and matrix degradation markers were not affected, this being a favorable finding regarding the potential of genipin for *in vivo* application. It appeared that 5 mM (and maybe lower concentrations) could induce relatively rapid crosslinking while leaving sub-populations of resident cells viable. In view of the clinical application of *in situ* crosslinking to arrest tendon tear propagation, it was deemed that the genipin concentration of 5 mM or slightly lower with an exposure of 72 h would be reasonable for *in vivo* studies.

It should be added that the said study focused only on acute and relatively rapid crosslinking effects, neither investigating whether long-term administration of genipin at lower doses (<1 mM) could possibly achieve a cumulative functional effect, nor observing the continuity of the documented effects. At a more basic level, so far the authors did not investigate how genipin crosslinks are actually formed or remain stable within the tissue, these aspects depending upon time and concentration [102].

## 6. Bone Regeneration

Chitosan respects the physiological bone formation and healing processes, and most importantly it enhances favorably the biochemical responses, owing to its inherent immunostimulating properties and susceptibility to lysozyme. Bone healing involves a sequence of events that should not be disturbed by the presence of a composite or scaffold. At the time of a fracture, the disruption of bone architecture and vascular network results in loss of mechanical stability and local decrease in oxygen and nutrients. The inflammatory response is accompanied by the activation of macrophages and infiltration of platelets that release various cytokines, which probably play a role in the initiation of the repair process by acting on various cells: post-fracture periosteal osteoprogenitor cells and osteoblasts differentiate to produce new bone. This process involves fibroblast growth factors and bone morphogenetic proteins. To provide crucial nutrient supplies to the cells, new blood vessels develop into the fracture callus. The matrix composed of various collagen isotypes develops, which may be important for presenting cytokines to receptive cells.

The chemical and technological versatility of chitosan enables researchers to prepare elaborated composites: for example, the research works on bone regeneration with the aid of bone cements have become more refined in terms of the effects of chitosan composites on the cells involved in the healing process. With the advent of nanotechnology the applications of fairly non-toxic nanocrystalline hydroxyapatite extends from bone repair and augmentation to the delivery of drugs, growth factors and genetic material to the bone: for this purpose, particles of uniform size with controlled morphology can be manufactured by using macromolecules as templates. A number of advantages have become evident, particularly when nano-hydroxyapatite is crystallized using biomimetic methods, or when the biopolymers are submitted to biomineralization. The hydroxyapatite nanoparticles influence favorably the morphology of attached cells, as a consequence of the adsorption of extracellular matrix proteins from serum, that in turn bind osteoblast precursors. Thus, an additional peculiarity of chitosan is emerging from most recent studies, namely the capacity to influence both the mineralization and the cell activity.

Chitosan, *N*-carboxymethyl chitosan, fibroin and poly(l-lactic acid) are at the basis of new strategies useful to stimulate stem cells to become osteoblasts, and to make co-cultures of osteoblasts and osteoclasts. With the aid of chitosan and sulfated chitosan, important advances have been made in the field of the delivery of human and recombinant bone morphogenetic proteins, in particular the morphogenetic protein-2 that exhibits a positive effect in every step of the bone regeneration. Again, the advances made in histology, cell culture, and cytology are accompanied by equally important contributions from material chemistry [103,104,105].

### 6.1. Enhanced Osteogenesis

The combined antibacterial efficacy and the modulation of the osteoblast behavior are very important for orthopedic applications. For example, Wu, F. *et al.* [106] loaded genipin crosslinked carboxymethylchitosan hydrogel with gentamycin and achieved enhanced adhesion, proliferation, and differentiation of osteoblasts besides full inhibition of *Staphylococcus aureus*. The degradation time of the CM-chitosan as well as the cellular responses depended on the genipin quantity. The loading of gentamycin increased of course the antibacterial efficiency, but it was also beneficial for the osteoblastic cell responses. Overall, the biocompatibility of the prepared hydrogel could be tuned by acting on the concentration of genipin and gentamycin, which interact with the available chemical groups of chitosan [106].

Bone defects surgically produced in sheep and rabbit models have been treated with freeze-dried modified chitosans because they promote direct endochondral ossification. Moreover, the pattern of bone regeneration has been studied in an osteoporotic experimental model with bone morphogenetic protein linked to chitosan [107]. The chitosan + collagen scaffolds had high proliferative effect if the degree of acetylation of chitosan was high, regardless of molecular weight. SEM demonstrated that MC3T3-E1 osteoblasts grew well on all tested scaffolds.

To provide a novel and effective drug delivery system that can enhance osteogenesis, Wang, G.C. *et al.* [108,109] evaluated the BMP-2 adsorption and release of bone morphogenetic protein-2 (BMP-2) on the superficial hydroxyapatite nanostructure of a coated GEN-chitosan that exhibited a loading efficiency of 65% (1.30 μg). The release of BMP-2 lasted for over 14 days in simulated body fluid, and induced an increase in alkaline phosphatase, indicative of osteogenic differentiation of seeded BMSCs. Hydroxyapatite + GEN-chitosan scaffolds also stimulated mRNA expression of osteogenic differentiation markers, namely osteopontin for three days, and osteocalcin for 14 days. Thus the superficial biomimetic HAp nanostructure within the composite scaffold promoted osteogenic differentiation *in vitro*. The hydroxyapatite nanostructure within the organic porous scaffold worked as a calcium source and absorption/release agent that suggested the design of bioactive scaffold for bone engineering. Nano-hydroxyapatite was included in GEN-chitosan films that were shown to be deprived of cytotoxicity against L929 cells [110].

Chitosan enhances bone and cartilage formation owing to its structural similarity to the extracellular matrix of bone cells. The pore sizes of the traditional scaffolds however, are seldom within the optimal range for cell ingrowth (100–400 μm), and the pores are not interconnected enough to allow cell infiltration. Moreover, high porosity and degradability of those chitosan scaffolds make them too weak for the purpose of bone repair. To qualify for these applications, membranes and scaffolds have to be stable in a wet environment, and should withstand simultaneously chemical and mechanical stresses, thus they need to be stabilized. Allowing cell infiltration and efficient nutrient and waste exchange has been an important goal in making good tissue scaffolds. Interconnected pores not only achieve this goal but also allow neovascularization that prevent the formation of a necrotic core in the scaffold. In practice, a microcomputer controls the movement of the robotic arm in *x* and *z* directions and the platform in y direction. The needle is raised 400 μm during each layer-step in the *z* direction. By raising the syringe needle one layer-step in the *z* direction, successive layers of hydrogel fibers are deposited onto previous layers in a 0°–90° pattern. According to the data obtained by Liu, I.H. *et al.*, osteoblasts secreted collagen-I in the first week and gradually differentiated into osteocytes; later on they expressed alkaline phosphatase that initiated the mineralization process by providing an alkaline environment and facilitating formation and nucleation of calcium phosphate in the GEN-chitosan 3D-plotted scaffolds [111]. In this respect it is interesting to note that the above mentioned article by Colosi *et al.* [62] contains valuable detailed information on a simplified technological approach to the manufacture of 3D-plotted scaffolds.

Loss of fibrous structure upon contact with aqueous solutions could limit practical utilization of 3D-plotted or electrospun chitosan nanofibers. To meet the demands for tissue engineering uses [112], post-electrospinning crosslinking may be performed to inhibit solubility and improve mechanical properties [113].

The invention by Lelkes and Frohbergh provided a scaffold comprising an electroprocessed, genipin-crosslinked mineralized chitosan nanofiber, definitely purified and capable of supporting the maturation of osteoblasts [114]. Osteoprogenitor cells, mesenchymal cells, stem cells, and osteocytes, are equally suitable. In this area of stimuli sensitive (smart) biomaterials that can facilitate regeneration of critical-size bone lesions, Frohbergh *et al.* [115] tested biomimetic scaffolds electrospun from chitosan expected to promote tissue repair in a critical size calvarial defect. Chitosan fibre mats are non-toxic and biocompatible, and therefore are convenient as filtration membranes and scaffolds for tissue engineering. They compared the *in vitro* ability of electrospun genipin-crosslinked chitosan to analogous scaffolds containing hydroxyapatite.

The cellular metabolic activity exhibited a biphasic behavior, indicative of initial proliferation followed by subsequent differentiation for all scaffolds. After three weeks in maintenance medium, ALP activity of mMSCs seeded onto GEN-chitosan + HAp scaffolds was approximately twice as much that of cells cultured on plain GEN-chitosan. Said mineralized scaffolds were also osseointegrative *in vivo*, as inferred from the enhanced bone regeneration in a murine model of critical size calvarial defects. Treatment of the lesions induced a 38% increase in the area of de novo generated mineralized tissue after three months, whereas plain scaffolds led to 10% increase. Mineralized scaffolds pre-seeded with mMSCs yielded 45% new mineralized tissue formation in the defects. The presence of HAp in the scaffolds significantly enhances their osseointegrative capacity: thus the mineralized GEN-chitosan may represents an unique biomaterial with possible clinical relevance for the repair of critical calvarial bone defects. In fact, autografts are seldom available, and alternative materials lead to poor integration with the host bone, owing to the absence of periosteum that contains osteoprogenitor cells and is crucial for growth and remodeling of bone tissue. The same authors [116] developed a one-step platform to electrospin nanofibrous scaffolds from chitosan, which also contain hydroxyapatite nanoparticles and are crosslinked with genipin, to stimulate osteoblast differentiation and maturation similar to the periosteum. The average fiber diameters of the electrospun scaffolds were 227 ± 154 nm as spun, and increased to 335 ± 119 nm after crosslinking with genipin. The Young's modulus of the composite fibrous scaffolds was 142 ± 13 MPa, which is similar to that of the natural periosteum [49].

Whilst genipin strongly improves the mechanical properties of composite rods, Pu *et al.* reported that the GEN-chitosan network made the hydroxyapatite composite rods much more stable than the controls against enzymatic depolymerization. The latter was tested with lysozyme for 72 h and the results indicated a weight loss rate of 4% for samples deprived of genipin, *versus* 0.5% for the genipin-containing samples. The bending strength and bending modulus of the crosslinked rods could reach 161 MPa and 7.2 GPa, respectively, with *ca.* 60% and 26% increase compared with un-crosslinked ones. Therefore, GEN-chitosan + hydroxyapatite composite rods with excellent mechanical properties are useful for internal fixation of bone fractures [117].

Rheological studies by Pandit *et al.* [118] demonstrated that the stiffness of hydrogels made of methylcellulose, chitosan and agarose increased upon crosslinking the chitosan with increasing amounts of genipin. Based on these results, gels crosslinked with 0.5% (*w*/*v*) genipin, having one third of the amino groups of chitosan crosslinked, exhibited a stiffness of 502 ± 64.5 Pa along with optimal characteristics to support bone regeneration. The gelling time decreased with increasing genipin concentrations. Again, these favorable chemical effects were accompanied by modulated cellular behaviors: in fact, proliferation of human umbilical vascular endothelial cells decreased by 10.7 times with increasing gel stiffness, in contrast to fibroblasts and osteoblasts, where it increased with gel stiffness by 6.37 and 7.8 times, respectively. Expression of differentiation markers by osteoblasts (osteocalcin, osteopontin and alkaline phosphatase) were significantly enhanced in the 0.5% (*w*/*v*) crosslinked gel, which also demonstrated enhanced mineralization by Day 25. Gels crosslinked with 0.5% (*w*/*v*) genipin still demonstrated significant bacterial inhibition [118].

Ge S.H. *et al.* explored the viability and differentiation of periodontal ligament stem cells on a nanohydroxyapatite-coated GEN-chitosan scaffold *in vitro* and *in vivo* [119]. Cell seeded scaffolds were used in a rat calvarial defect model, and new bone formation was assessed by hematoxylin and eosin staining at 12 weeks postoperatively. When seeded on said scaffolds the stem cells exhibited significantly greater viability, and up-regulated the bone-related markers to a greater extent than for controls, thus the calvarial bone repair was obtained.

### 6.2. Technical Advances, Novel Know-How and Improvements

**Figure 5:**
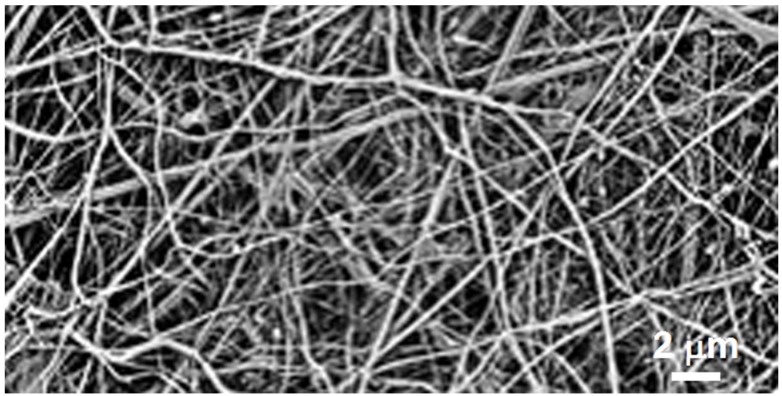
Typical genipin-crosslinked chitosan fiber mat obtained from electrospun chitosan mat subsequently treated with genipin: the nanofibrous form is preserved.

Genipin spontaneously crosslinks chitosan gels, microspheres, and fibers (Figure 5), even in the presence of PVA, PVP, PEO, fibroin or gelatin. Data by Nwosu *et al.* indicate that being able to improve the stability of smart GEN-chitosan + PVP with solely physical manipulations without altering the initial chemical composition is an aspect of potential medical applications, for example, in wound dressing and drug delivery [120].

GEN-Chitosan + bioglass + PVP scaffolds were prepared by Yao Q.Q. *et al.* [121]. Improved resistance to enzymatic degradation of the scaffolds was obtained while enhancing biocompatibility and porosity of the bioglass scaffolds for good adhesion and proliferation of pre-osteoblasts MC3T3-E1 cells. The latter within the scaffolds showed well stretched F-actin bundles after four-day incubation. These structures, known as tunneling nanotubes, could mediate the intercellular transfer of organelles, plasma membrane components and cytoplasmic substances. Furthermore, those scaffolds qualified for the controlled delivery of the antibiotic vancomycin.

While several laboratories reported that that 0.025% genipin is sufficient to fully crosslink chitosan, Austero *et al.* [122] and Donius *et al.* [123] used 0.10% for two reasons: first, excess of the crosslinker was added to react with all the amino groups of chitosan; second, at concentrations less than 0.10%, the mats were soluble in acidic and neutral solutions. The 0.10% concentration enabled to make improvements in stability and versatility. Typical values for chitosan-genipin fiber mats were fiber diameter 176 ± 106 nm; fiber mat porosity 55.5% ± 6.8%; fiber-fiber contacts per unit volume 4.45 μm^−3^ [122,123]. The same research team [124] manufactured fibrous chitosan + hydroxyapatite composite scaffolds with 1 and 10 wt % mineral contents by electrospinning. The fibers, crosslinked with genipin, contained crystalline hydroxyapatite at 10% additive. Electrospun fibers had diameters 122–249 nm, in the range of those of fibrous collagen found in the extracellular matrix of bone. Young’s modulus and ultimate tensile strength of the various crosslinked composite were in the range 2–15 MPa. Osteocytes seeded onto the mineralized fibers demonstrated good biocompatibility.

In a dynamic perfusion culture apparatus the flow rate of a culture medium through a chitosan scaffold influenced cell proliferation and expression of bone marker genes. The feasibility of culturing osteoblast-like MG-63 cells on chitosan + genipin scaffolds was demonstrated by Su, W.T. *et al.* [125] who confirm that flow perfusion cultures can improve cellular distribution and abundance in porous scaffolds. In fact, mineralized tissue is distributed throughout the entire area of the scaffolds cultured under flow perfusion; on the contrary on scaffolds cultured under static conditions fewer mineral depots are detected. These results suggest that osteoblast-like MG-63 cells seeded in chitosan scaffolds produce more calcium and phosphate in dynamic culture. In the latter, cells seeded into a scaffold receive mechanical stimulation provided by the mobile fluid, and undergo multilayered 3D growth and organization, thereby enhancing bone-related gene and phenotypic expression [126,127]; thus collagen-I and OCN gene expression are higher in dynamic culture than in static culture. A continuously pumping gas-permeable silicon tube allows for optimal exchange of gases, so that the O_2_ partial pressure in the medium is higher than in a conventional culture plate. These results are in agreement with earlier articles, which reported that cultivation of osteoblast-like cells and rat bone marrow stem cells on 3D scaffolds in a perfusion culturing system enhances growth, differentiation, and mineralized matrix production *in vitro*.

Fully characterized scaffolds in terms of porosity, pore size, swelling, wettability, compressive strength, and mass loss were seeded with human mesenchymal stem cells and evaluated with respect to osteogenic differentiation with incubation time [128]. Experimental groups included GEN-chitosan + β-tricalcium phosphate that displayed interconnected honeycomb-like microstructures with porosity >65%. There was linear dependence of both water contact angle and pore size with crosslinker concentration. The metabolic activity of hMSCs seeded in those scaffolds was significantly higher than for controls, as well as their mineralization after 21 days of incubation in osteogenic medium.

Guided bone regeneration membranes prevent soft tissue infiltration into the graft space during dental interventions that involve bone grafting. Chitosan materials have shown promise in this area, owing to their biocompatibility and predictable biodegradability, but longer degradation time periods are needed for clinical applications. Chitosan membranes were electrospun using chitosan (70% deacetylated, 312 kDa, 5.5% *w*/*v*), with or without the addition of 5 or 10 mM genipin, in order to extend the degradation to meet the clinical time of four months [129]. Genipin addition resulted in median fibre diameters 144 nm, 154 nm respectively for 5 mm and 10 mm crosslinked, and 184 nm for uncrosslinked samples. The ultimate tensile strength of the mats was increased by 165% to 32 MPa with 10 mm crosslinking as compared to the uncrosslinked mats. Genipin-chitosan samples exhibited only 22% degradation based on mass loss, as compared to 34% for uncrosslinked mats at four months *in vitro*. Therefore electrospun chitosans may benefit from the reaction with genipin and can meet clinical degradation time frames for guided bone regeneration.

## 7. Conclusions

Recent works have produced a wealth of data on the advantages offered by new physical forms of chitosan stabilized with genipin [95,130,131,132,133,134,135,136,137,138,139,140]. The advances made become quite noticeable when the works dated 2010–2015 (amounting to >82% of the bibliography below) are compared to some most significant key articles [5,11,27,41,50,141,142,143,144,145,146] published in the 2000–2005 quinquennium.

The research topics currently considered span from the extraction of genipin to the preparation of advanced devices suitable for tissue engineering. The biochemical processes adopted in the preparation of the pigment aim at drastic improvement of the process yields, and cost abatement. Likewise, novel views on the manipulation of human cells permit to refine the preparation of scaffolds intended for the maximum performance: for example in the current year electrospun chitosan layers have been put on the market by Advanced BioMatrix, San Diego, CA, USA, to complete a catalog of analogous products based on collagen, gelatin, alginate, hyaluronan and more, and intended for cell culture and tissue engineering.

After the layer-by-layer scaffolds, today it is possible to generate functional biomimetic surfaces with tuned mechanical and biochemical properties. Simplified technological approaches to the manufacture of 3D-plotted scaffolds are providing exciting developments.

Authors unanimously recognize that electrospun nanofibrous chitosan scaffolds crosslinked with genipin are most attractive for tissue engineering. Nevertheless, the preparations described in the above cited articles most often omit indispensable details. It should be underlined that minor amounts of genipin are necessary when the latter is the ingredient of a scaffold, and that the final scaffold is expected to be light blue instead of black as shown in some illustrations. Besides that, it is becoming mandatory to express the calculations in terms of molar ratios between the components of a scaffolds whose quality has to be assessed by advanced instrumental analyses if they are expected to be recognized for purity, functionality and consistency.
